# Relation between Acute GVHD and NK Cell Subset Reconstitution Following Allogeneic Stem Cell Transplantation

**DOI:** 10.3389/fimmu.2016.00595

**Published:** 2016-12-22

**Authors:** Evelyn Ullrich, Emilia Salzmann-Manrique, Shahrzad Bakhtiar, Melanie Bremm, Stephanie Gerstner, Eva Herrmann, Peter Bader, Petra Hoffmann, Ernst Holler, Matthias Edinger, Daniel Wolff

**Affiliations:** ^1^LOEWE Center for Cell and Gene Therapy, Goethe University, Frankfurt, Germany; ^2^Division for Stem Cell Transplantation and Immunology, Department for Children and Adolescents Medicine, Hospital of the Goethe University Frankfurt, Frankfurt, Germany; ^3^Institute of Biostatistics and Mathematical Modeling, Johann Wolfgang Goethe University, Frankfurt, Germany; ^4^Department of Internal Medicine III, University Hospital Regensburg, Regensburg, Germany; ^5^Regensburg Center for Interventional Immunology (RCI), University of Regensburg, Regensburg, Germany

**Keywords:** NK cell, NK cell subset, GVHD, SCT, immune reconstitution

## Abstract

One of the major challenges of allogeneic stem cell transplantation (allo-SCT) is to reduce the risk of graft-versus-host disease (GVHD) while boosting the graft-versus-leukemia (GVL) effect. The reconstitution of natural killer (NK) cells following allo-SCT is of notable interest due to their known capability to induce GVL without GVHD. Here, in this study, we investigate the association between the incidence and severity of acute graft-versus-host disease (aGVHD) and the early reconstitution of NK cell subsets following allo-SCT. We analyzed 342 samples from 107 patients using flow cytometry, with a focus on immature CD56^high^ and mature cytotoxic CD56^dim^ NK cells. Longitudinal analysis of immune reconstitution after allo-SCT showed that the incidence of aGVHD was associated with a delayed expansion of the entire NK cell population, in particular the CD56^high^ subset. Notably, the disturbed reconstitution of the CD56^high^ NK cells also correlated with the severity of aGVHD.

## Introduction

Allogeneic stem cell transplantation (allo-SCT) often remains the only curative treatment for hematological disorders. However, its success is frequently limited by acute and chronic graft-versus-host disease (GVHD), causing significant morbidity and mortality (1). One of the major challenges of allo-SCT is to reduce the incidence and severity of GVHD while boosting the graft-versus-leukemia (GVL) effect. In the setting of allo-SCT, the reconstitution of natural killer (NK) cells is of notable interest due to their known capability to induce GVL without GVHD (2). NK cells are known to play an important role in innate and adaptive immunity as well as in immunotherapeutic approaches (3–5). Recently, we could demonstrate that NK cells gain cytotoxic and cytokine producing functions early during hematopoietic immune reconstitution following autologous SCT (6). In addition to clinical studies, it has been shown in animal models that IL-2-activated NK cells may efficiently prevent or even reduce GVHD without any adverse impact on their important GVL effect (7–9). In humans, KIR mismatch in haploidentical BMT in the GVH direction reduced the risk of GVHD (2, 10), and adaptive cell therapies using expanded NK cells have been established for different malignancies and for the relapse of leukemia (3, 5, 11, 12).

However, NK cells are a heterogeneous population that can be classified into phenotypically and functionally distinct subsets (9, 13, 14). Recently, we demonstrated in a mouse allo-SCT and GVHD model that the mature subset of fully cytotoxic NK cells specifically mediates both the antitumor (GVL) and the GVHD-reducing effects (9).

In line with this finding, it is well known that NK cell reconstitution following allo-SCT correlates with higher numbers of immature CD56^high^CD16^dim^ NK cells that further differentiate into cytotoxic CD56^dim^CD16^high^ NK cells (15–17). While immune reconstitution *per se* has been shown to be influenced by the occurrence of acute graft-versus-host disease (aGVHD) and chronic GVHD and the need for immunosuppressive treatment (18), it is not yet completely known to which extent early NK cell reconstitution is influenced by the occurrence of aGVHD.

Here, in our single-center immune monitoring study performed at the University Hospital Regensburg, we investigated the possible correlation between the regeneration of NK cell subsets and the incidence of aGVHD during the first 200 days following allo-SCT, with a focus on immature NK cells (CD14^−^CD3^−^CD56^high^CD16^dim^), mature cytotoxic NK cells (CD14^−^CD3^−^CD56^dim^CD16^high^), and the ratio of these two populations (CD56^dim^:CD56^high^).

## Materials and Methods

### Study Design and Sample Collection

This study was approved by the Ethics committee of the University Regensburg, Germany (approval no. 02/220) and carried out in accordance to the Declaration of Helsinki. All subjects gave written informed consent in accordance with the Declaration of Helsinki. In this study, data were collected from 2009 to 2012. In sum, 107 patients were included into this study. Patient characteristics are described in Table 1. HLA typing was always performed with the same strategy using high resolution HLA-A, B, DR, and DQ for sibling and additional HLA-C typing for unrelated donors according to the standard of the European Federation for Immunogenetics.

**Table 1:** **Patient’s characteristics**.

Characteristics	Total		With acute graft-versus-host disease (aGVHD)		Without aGVHD		*p*
	*n*	%	*n*	%	*n*	%	
Patients	107	100	62	100	45	100	
**Age at SCT**							*1*
Median (range), years			52.7 (17.5–70.1)		50.3 (24.3–70.7)		
**Sex**							*0.013*
Female	37	35	15	24	22	49	
Male	70	65	47	76	23	51	
**Disease**							*0.133*
ALL	10	9	8	13	2	4	
AML	45	42	23	37	22	49	
CLL	5	5	5	8	0	0	
CML	1	1	1	2	0	0	
Lymphoma	10	9	4	6	6	13	
MDS	12	11	9	15	3	7	
MM	19	18	10	16	9	20	
Other	5	5	2	3	3	7	
**Donor relation**							*0.191*
Unrelated	77	72	48	77	29	64	
Related sibling	30	28	14	23	16	36	
**Donor HLA**							*0.443*
Identical	88	82	49	79	39	87	
Mismatched	19	18	13	21	6	13	
**Stem cell source**							*0.627*
BM	8	7	4	6	4	9	
PBSC	98	92	57	92	41	91	
CB	1	1	1	2	0	0	
**Conditioning**							*0.130*
RIC	86	80	46	75	40	89	
MA	20	19	15	25	5	11	
**Serotherapy**							
ATG	58	54	35	56	23	51	
**Graft-versus-host disease (GVHD) prophylaxis**							*0.927*
CsA + MTX	84	79	48	77	36	80	
CsA + MMF	19	18	12	19	7	16	
Other	4	4	2	4	2	4	

*There were 107 patients included in this study. The distributions are shown for the underlying diseases, age, stem cell source, serotherapy, and GVHD prophylaxis. The p-value was determined using the Fisher-Exact test or the Mann–Whitney test, as appropriate*.

*SCT, stem cell transplantation; ALL, acute lymphocytic leukemia; AML, acute myeloid leukemia; CLL, chronic lymphocytic leukemia; CML, chronic myeloid leukemia; MDS, myelodysplastic syndrome; MM, multiple myeloma; BM, bone marrow; PBSC, peripheral blood stem cells; CB, cord blood; RIC, reduced-intensity conditioning; MA, myeloablative; ATG, anti-thymocyte globulin; CsA, cyclosporin A; MTX, methotrexat; MMF, mycophenolate mofetil; p, p-value. p-Value was performed using the Fisher-Exact test or Mann–Whitney test as appropriate*.

Patient blood samples were collected prior to and following allo-SCT at different time points during regular outpatients visits. The frequency of sample collection was part of the individual follow-up plan for each patient at fixed time points and additionally when specific events occurred during the first 200 days after their treatment by allo-SCT. Exclusion criteria were the development of chronic GVHD or an overlap syndrome with signs of both acute and chronic GVHD.

Peripheral blood mononuclear cells of each sample were freshly isolated by density gradient centrifugation (Pancoll human, Pan-Biotech) and within 24 h analyzed by flow cytometry (FACS). Samples of healthy controls were processed in exactly the same way as patient samples. The following antibodies were used: CD3-FITC (SK7), CD16-PE (B73.1), CD14-PerCP (MΦP9), and CD56-APC (N-CAM 16-2), all from BD Biosciences. The focus of this analysis was on immature CD56^high^ and mature cytotoxic CD56^dim^ NK cells. At each time point, patients were considered to be “with aGVHD” or “without aGVHD” based on clinical findings. The evaluation of aGVHD was performed on a weekly basis applying the modified Keystone and NIH criteria (19, 20). All patients suffering from aGVHD, at least at one sample drawing, were included in the aGVHD group, those without any signs of aGVHD at any sampling time point were included in the group without aGVHD.

### Statistical Considerations

First, patient characteristics were summarized (Table 1) and the Fisher-Exact test or Mann–Whitney test was used as appropriate for comparisons. Classification of GVHD was performed according to the NIH consensus criteria (20). Additionally, the cumulative incidence (CI) of aGVHD has been assessed considering relapse and death without relapse as competing event. In the same way, we analyzed the CI considering the grade of aGVHD.

With the aim to perform a longitudinal analysis of the NK cell numbers associated with aGVHD development and severity, a mixed effect regression model with a linear spline model was used. The reconstitution profile analyzed in this study comprised the dynamics of absolute values of NK cells, CD56^high^ cells, CD56^dim^ cells, and the ratio CD56^dim^ cells:CD56^high^ cells within the first 200 days after allo-SCT. Patients were previously classified into two groups according to their clinical and laboratory findings of aGVHD as described before. All measurements from patients who did not develop any signs of aGVHD at any collection time point measured during 200 days following allo-SCT were included in the longitudinal analysis. For patients who developed aGVHD during the 200 days period after allo-SCT, data measured from allo-SCT until achievement of aGVHD remission were included in the analysis. For each NK subset, the incidence of aGVHD and the severity of aGVHD were considered separately to be dependent variables. Furthermore, to evaluate the effect of steroid administration on NK cell reconstitution, the aGVHD patients were classified regarding GVHD stage and steroid treatment (ST) at each observation. Afterward, linear spline regression models were fitted separately for each NK cell subpopulation.

To optimize the residual distribution of the regression analysis, cell counts of NK subsets and the analyzed ratio were previously log_10_-transformed. Note that the statistical regression methods used here account for repeated measurements at different time points.

All statistical tests were two-sided with a significance level of 5% representing the 95% confidence interval. Data analysis was performed using the R software for statistical computing, version 3.1.1 (R Foundation for Statistical Computing, Vienna, Austria, http://www.R-project.org/). Figure 3 was generated using GraphPad Prism 6 software, version 6.0.4.

## Results

### Patient Characteristics

A total of 342 samples have been analyzed from 107 patients (78 prior to SCT, 264 after SCT), 37 (35%) females, and 70 (65%) males, were included in this study. Fifty-five (51%) patients suffered from acute leukemia, 19 (18%) from multiple myeloma, 12 (11%) from MDS, 10 (9%) from lymphoma, 6 from chronic leukemia (6%) and 5 from other diseases (5%), as shown in Table 1. The majority of the patients underwent HLA-identical allo-SCT (*n* = 88, 82%). Thirty patients had related sibling donors (28%), 77 had unrelated donors (72%). The stem cell source was peripheral blood stem cells in 98 patients (92%), bone marrow (BM) in eight patients (7%), and cord blood in one patient (1%). A reduced-intensity conditioning (RIC) regimen was administered in 86 patients (80%), and 21 patients were treated using myeloablative regimens (20%). Fifty-eight patients received anti-thymocyte globulin treatment (54%). GVHD prophylaxis consisted of CSA + MTX in 84 patients (79%). Others received immunosuppressive monotherapy or individual combination treatments (see Table 1). While 45 (42%) patients did not show signs of aGVHD, 62 (58%) patients developed aGVHD (grade 1: *n* = 22, grade 2: *n* = 21, grade 3: *n* = 14, grade 4: *n* = 5) at a median of day 28 (11–182) after allo-SCT. Symptoms that led to the classification of aGVHD and/or cGVHD were defined according to the NIH criteria as recently published (20).

No major difference in age, underlying disease, donor type, stem cell source, HLA match, conditioning regimen, and GVHD prophylaxis between the GVHD group and the group of patients without GVHD was observed. The only parameter contributing to a significant difference between the two mentioned groups was gender with a higher proportion of male patients in the GVHD group (*p* = 0.013).

The median age of the GVHD group was 52.7 years (17.5–70.1) and of the group of patients without signs of aGVHD 50.3 years (24.3–70.7). In both groups, the majority of patients suffered from AML (49%). Additional underlying diseases are indicated in Table 1.

The median survival of the group that did not develop aGVHD was 34.1 months (ranging from 0.9 to 93.4 months), whereas the median survival of the GVHD group was 21.6 months (ranging from 1.2 to 65.7 months). The estimated CI of overall aGVHD was 57.9% (95% CI: 48.5–67.30%); the estimated CI for aGVHD grade I was 18.88% (95% CI: 11.42–26.34%), 19.73% (95% CI: 12.2–27.38%) for aGVHD grade II, 13.19% (95% CI: 6.75–19.64%) for aGVHD grade III, and 3.76% (95% CI: 0.68–8.78%) for severe aGVHD grade IV (Figure S1 in Supplementary Material). A critical phase with very high numbers of incidences occurred between days +25 and +50 after allo-SCT.

### NK Cell Subsets

**Figure 1:**
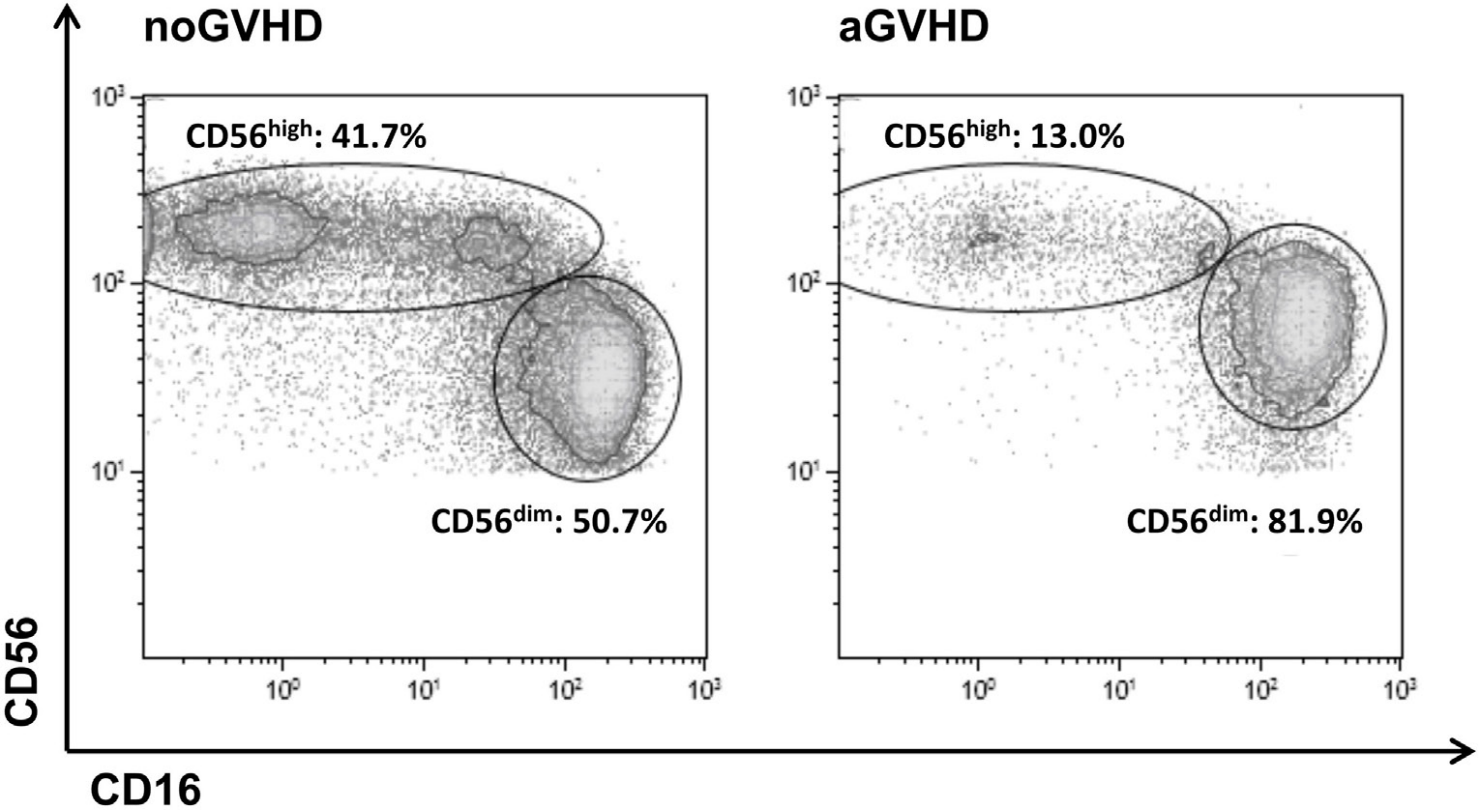
**Definition of the CD56^high^CD16^dim^ and CD56^dim^CD16^high^ natural killer (NK) cell subsets**. Representative FACS dot plots of all CD56^+^CD3^−^CD14^−^ NK cells from a patient on day 24 post-allogeneic stem cell transplantation without (left) or from a patient on day 28 post-allo-SCT with (right) development of acute graft-versus-host disease.

A density plot gated on all CD56^+^CD3^−^CD14^−^ NK cells further distinguished two major subsets including immature CD56^high^CD16^dim^ (hereinafter referred to as CD56^high^) and mature cytotoxic CD56^dim^CD16^high^ NK cells (hereinafter referred to as CD56^dim^). In healthy adult controls, only 12.5 ± 9.6% of all NK cells belong to the immature CD56^high^ NK population whereas the majority of 83.1 ± 12.2% represents mature CD56^dim^ NK cells that coexpress CD16 (data not shown). Importantly, in our study cohort, there was no significant preexisting difference in NK cell subset distribution prior to allo-SCT among patients that later developed GVHD or not (Figure S2 in Supplementary Material). The appearance of immature CD56^high^ NK cells that do not yet express the Fcγ-receptor CD16 on their surface is indicative of an efficient immune reconstitution post-allo-SCT. In this study, following allo-SCT, the immature subset of CD56^high^ NK cells was highly increased and reached up to 45% of all NK cells in patients without aGVHD, but was significantly reduced in patients who developed aGVHD (Figure 1).

### Impact of aGVHD on NK Cell Reconstitution

Next, we addressed a possible impact of aGVHD on the NK cell numbers and early NK cell reconstitution throughout an observation period of 200 days after allo-SCT. Patients without any signs of aGVHD during the whole observation period had considerably higher NK cell numbers than patients who developed aGVHD, with significant differences in the CD56^high^ NK cell subset (Figure S2 in Supplementary Material). In contrast, there was only a trend of reduced numbers of CD4^+^ T cells around week 12 post SCT, but overall numbers of CD3^+^ T cells did not significantly differ in GVHD patients compared to patients without GVHD following allo-SCT (Figure S3 in Supplementary Material).

Moreover, as shown in Table 2, the longitudinal analysis of the logarithmically transformed numbers of the different NK cell subsets revealed a negative association between the occurrence of aGVHD and the early expansion of the total NK cell population (*p* = 0.06), and in particular the CD56^high^ NK cell subset (*p* = 0.009). In contrast, there was no significant correlation between the CD56^dim^ NK cell subsets or the calculated ratio of CD56^dim^:CD56^high^ NK cells and aGVHD (Table 2). Furthermore, a significant inverse correlation between the severity of aGVHD and the frequency of CD56^high^ NK cells during aGVHD could be demonstrated by the linear spline mixed effect model (Table 3).

**Table 2:** **Impact of aGVHD on immune reconstitution of NK cell subsets after allogeneic stem cell transplantation (allo-SCT)**.

	Beta (SE)	*p*-Value
**NK cells**		
aGVHD	−0.146 (0.08)	0.061
**CD56^high^**		
aGVHD	−0.219 (0.08)	0.009
**CD56^dim^**		
aGVHD	−0.114 (0.09)	0.194
**Ratio**		
aGVHD	0.106 (0.07)	0.158

*The table summarizes the results of linear spline regression models with mixed effects analyzing the influence of aGVHD on each NK cell subset during the first 200 days after allo-SCT. The beta coefficients are given in log10 scale due to previous log10-transformed NK cell subsets*.

*NK cells, natural killer cells; CD56^high^, immature CD14^−^CD3^−^CD56^high^CD16^dim^ NK cells; CD56^dim^, mature CD14^−^CD3^−^CD56^dim^CD16^high^ NK cells; Ratio, ratio CD56^dim^:CD56^high^; aGVHD, acute graft-versus-host disease; Beta, beta coefficient*.

**Table 3:** **Impact of the aGVHD grade on the number of CD56^high^ natural killer (NK) cells**.

CD56^high^	Beta (SE)	*p*-Value
aGVHD grade 1	0.007 (0.11)	0.94
aGVHD grade II	−0.28 (0.10)	0.008
aGVHD grade III	−0.48 (0.12)	<0.001
aGVHD grade IV	−0.43 (0.16)	0.009

*The table shows the beta coefficients and corresponding p-values for each aGVHD grade with respect to the reference category without aGVHD from a mixed effect regression with a linear spline model. Patients with aGVHD grade III or IV showed a significantly lower frequency of CD56^high^ NK cells compared to patients with aGVHD grade I or II*.

*aGVHD, acute graft-versus-host disease; Beta, beta coefficient*.

**Figure 2:**
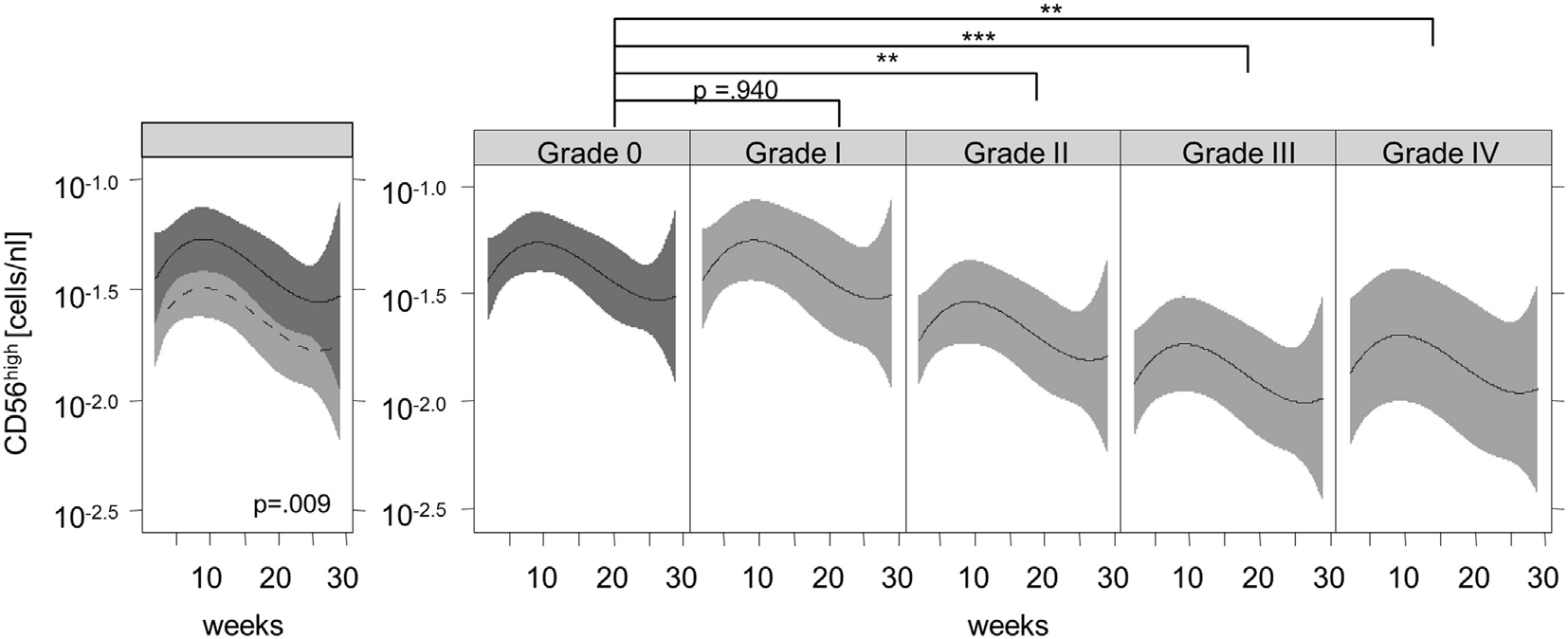
**Association of acute graft-versus-host disease (aGVHD) and the immune recovery of CD56^high^ natural killer (NK) cells**. Lines represent the back-transformed CD56^high^ NK cell numbers estimated from the model. The solid line shows the cell recovery in patients without aGVHD, and the dashed line indicates patients with aGVHD and the corresponding 95% confidence interval (left graphic). A fitted model considering aGVHD severity illustrates a significant correlation between aGVHD grade and the lower recovery of CD56^high^ NK cells during aGVHD (right graphic). **p < 0.01, ***p < 0.001.

Figure 2 shows the recovery of the CD56^high^ NK subset in patients without signs of aGVHD (solid black line) compared to patients with aGVHD (dashed black line) and their corresponding 95% confidence intervals. We observed significantly lower numbers of CD56^high^ NK cells in the aGVHD group, suggesting that aGVHD might cause impaired CD56^high^ NK cell regeneration in the early phase after allo-SCT.

**Figure 3:**
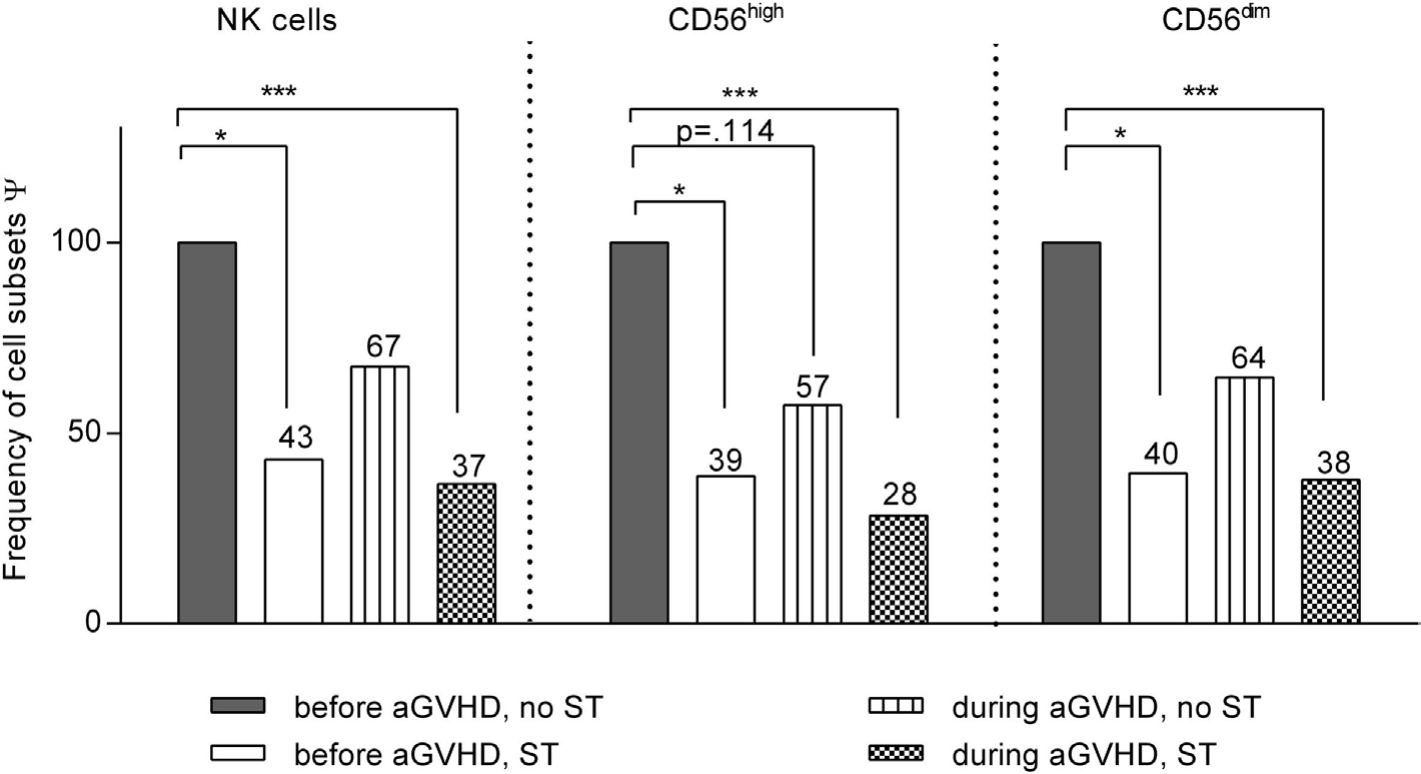
**Impact of steroid application on natural killer (NK) cell subset distribution**. Linear spline model including all patients that developed acute graft-versus-host disease at least at one measurement during the entire observation period after allogeneic stem cell transplantation (allo-SCT). All NK cell data were classified into four categories according to the stage of graft-versus-host disease (GVHD) (before or during/after GVHD) and the treatment with steroids [steroid treatment (ST) or no ST]. The bars illustrate the frequency of each NK cell subset related to the respective estimate regression coefficients for that specific subset compared to that of patients before GVHD development and without ST (100% reference). *p < 0.05, and ***p < 0.001. Ψ related to estimated coefficients.

Furthermore, the reconstitution of the CD56^high^ subpopulation was analyzed with regard to the severity of aGVHD. We observed a significant correlation between the grade of aGVHD and the degree of disturbance of CD56^high^ NK cell reconstitution (Figures 2 and 3). There was almost no difference in NK cell immune reconstitution between patients with grade I GVHD and those without GVHD. In contrast, patients with severe grade of aGVHD (III–IV) showed lower numbers of CD56^high^ NK cells in comparison to patients with milder forms of aGVHD. In summary, not only the occurrence but also the severity of aGVHD correlated with a delayed reconstitution of the CD56^high^ NK cell subset.

### Impact of Steroids

To elucidate the impact of steroid treatment (ST) on NK cell recovery, the frequencies of the different NK cell subpopulations have been calculated for all patients who developed aGVHD at least at one sampling time during the entire observation period of 200 days (Figure 3). All data on the NK cell numbers measured in these GVHD patients have been classified and analyzed according to the stage of disease at the given time point, as being obtained either before or during development of GVHD and with or without ST. Reasons for ST without occurrence of GVHD were in most cases inappetence, nausea, gastroenteritis, and weakness treated with moderate dosing of prednisolone. Only few patients received high dose prednisolone due to, e.g., a severe capillary leak, vasculitis, or ARDS. GVHD patients were also treated with prednisolone (1 mg/kg BW).

Figure 3 and Table S1 in Supplementary Material show the results of the linear regression model with smoothing spline performed with the above mentioned disease and treatment classifications of all GVHD patients. All results are shown in relation to measurements at time points without signs of aGVHD and without ST that served as base line (=100%) of each individual patient. In sum, we observed an additive impact of steroids and aGVHD on reconstitution of NK cell subsets. Of note, based on these analysis, we can exclude that the before mentioned negative impact of aGVHD on the CD56^high^ subpopulation recovery could be completely explained by ST.

## Discussion

The complex interplay of NK cells and NK cell subsets with other immune cells in the development of aGVHD following allo-SCT has mainly been investigated in different animal models, but only very few human studies have addressed NK cells thus far (8, 9, 15–17, 21).

However, following allo-SCT, NK cells are among the first lymphocytes to reconstitute and reach normal numbers after 1–4 months, independent of the stem cell source (17, 19, 22, 23).

In line with our previous preclinical observation that NK cell maturation was impaired in mice suffering from aGVHD and that only the subset of mature CD11b-expressing NK cells efficiently protects from developing aGVHD in a mouse model of allogeneic BMT (9), Podgorny et al. investigated a large patient cohort and observed a reduction in the number of total and cytotoxic NK cells during aGVHD (15). This effect might be due to a GVHD-induced destruction of the hematopoietic niche in the BM as demonstrated by the effect on B cell reconstitution (24, 25). However, this study did not address the impact of aGVHD on early NK cell reconstitution.

Notably, NK cells play an important role in the early innate immune defense, and a recent report has shown an association of the reduced number of total and CD56^high^ NK cells with postengraftment infection rates after allo-SCT (26).

In our study, we analyzed 342 samples of 107 patients before and after allo-SCT for a possible association between aGVHD and NK cell numbers including subset composition at different time points during the first 200 days following allo-SCT.

To our knowledge, our data are the first to demonstrate that aGVHD correlates not only with reduced numbers of total NK cells but specifically with the disturbed regeneration of CD56^high^ NK cells early after allo-SCT. Patients without any aGVHD during the whole observation period had a higher number of NK cells and specifically the CD56^high^ NK cell subset than patients who developed aGVHD (Table 2; Figure S2 in Supplementary Material), while T cell numbers did not significantly differ (Figure S3 in Supplementary Material).

Furthermore, we performed a longitudinal analysis to investigate the impact of aGVHD on early NK cell reconstitution (Tables 2 and 3). Remarkably, we found a significant correlation between the severity of aGVHD and the degree of disturbance of CD56^high^ NK cells during aGVHD (Figure 2; Table 3). As described before (15), we also found a non-significant trend toward a decrease in total and cytotoxic CD56^dim^ NK cells. A possible explanation for differences in comparison to other studies might be the study design and classification of patients according to severity of aGVHD (27).

In summary, our data suggest a negative impact of aGVHD on early NK cell reconstitution and maturation resulting in a disturbed NK cell subset composition. Interestingly, these developments seemed to take place regardless of the immunosuppressive effect of ST (Figure 3; Table S1 in Supplementary Material). Of note, it cannot be excluded that negative effects of steroids on NK cell reconstitution might influence the risk of GVHD post-allo-SCT.

The most probable cause for the delayed NK recovery is a lymphocyte maturation defect in the BM niche. However, it still remains unclear if the reduced number of NK cells is the underlying cause or a consequence of aGVHD. Mouse experiments suggest that mature NK cells might be capable of protecting from aGVHD by inhibition of allogeneic T cell proliferation, either directly by perforin-mediated killing of T cells or indirectly by lysis of antigen presenting host cells that support T cell lysis (8, 9). However, it should be taken into account that this effect was observed in a HLA-mismatched situation while the presented data relate to HLA-matched transplantation. In patients, a significant decline in the number of CD56^high^ NK cells early after allo-SCT might even be a predictive parameter for the development of aGVHD. However, this statement remains hypothetical as we did not have enough measurements of the patients CD56^high^ NK cell numbers shortly before the appearance of first GVHD signs that allowed the statistical evaluation in this context.

Therefore, further studies are needed to unravel the value of NK cell numbers as biomarker of aGVHD as well as the pathophysiology of the impaired NK cell regeneration that might be caused by a GVHD-induced destruction of the hematopoietic niche in the BM or a lack in hematopoietic growth factors.

## Author Contributions

EU, SB, MB, and SG, performed the experiments, analysed, and prepared figures; ES-M, EH, and MB performed the bioinformatics, statistical analysis, and generated figures; PH, EH, ME, and DW designed the study; EU, PB, PH, EH, ME, and DW discussed the results; EU and DW evaluated data and wrote the manuscript with support of all co-authors.

## Conflict of Interest Statement

The authors declare that the research was conducted in the absence of any commercial or financial relationships that could be construed as a potential conflict of interest.

## References

[1] Ferrara JL Levine JE Reddy P Holler E. Graft-versus-host disease. Lancet (2009) 373(9674):1550–61. 10.1016/S0140-6736(09)60237-3 19282026 PMC2735047

[2] Ruggeri L Capanni M Urbani E Perruccio K Shlomchik WD Tosti A Effectiveness of donor natural killer cell alloreactivity in mismatched hematopoietic transplants. Science (2002) 295(5562):2097–100. 10.1126/science.1068440 11896281

[3] Terme M Ullrich E Delahaye NF Chaput N Zitvogel L. Natural killer cell-directed therapies: moving from unexpected results to successful strategies. Nat Immunol (2008) 9(5):486–94. 10.1038/ni1580 18425105

[4] Vivier E Tomasello E Baratin M Walzer T Ugolini S. Functions of natural killer cells. Nat Immunol (2008) 9(5):503–10. 10.1038/ni1582 18425107

[5] Childs RW Carlsten M. Therapeutic approaches to enhance natural killer cell cytotoxicity against cancer: the force awakens. Nat Rev Drug Discov (2015) 14(7):487–98. 10.1038/nrd4506 26000725

[6] Jacobs B Tognarelli S Poller K Bader P Mackensen A Ullrich E. NK cell subgroups, phenotype, and functions after autologous stem cell transplantation. Front Immunol (2015) 6:583. 10.3389/fimmu.2015.00583 26635797 PMC4657185

[7] Asai O Longo DL Tian ZG Hornung RL Taub DD Ruscetti FW Suppression of graft-versus-host disease and amplification of graft-versus-tumor effects by activated natural killer cells after allogeneic bone marrow transplantation. J Clin Invest (1998) 101(9):1835–42. 10.1172/JCI1268 9576746 PMC508768

[8] Olson JA Leveson-Gower DB Gill S Baker J Beilhack A Negrin RS. NK cells mediate reduction of GVHD by inhibiting activated, alloreactive T cells while retaining GVT effects. Blood (2010) 115(21):4293–301. 10.1182/blood-2009-05-222190 20233969 PMC2879101

[9] Meinhardt K Kroeger I Bauer R Ganss F Ovsiy I Rothamer J Identification and characterization of the specific murine NK cell subset supporting graft-versus-leukemia- and reducing graft-versus-host-effects. Oncoimmunology (2015) 4(1):e981483. 10.4161/2162402X.2014.981483 25949862 PMC4368119

[10] Giebel S Locatelli F Lamparelli T Velardi A Davies S Frumento G Survival advantage with KIR ligand incompatibility in hematopoietic stem cell transplantation from unrelated donors. Blood (2003) 102(3):814–9. 10.1182/blood-2003-01-0091 12689936

[11] Miller JS Soignier Y Panoskaltsis-Mortari A McNearney SA Yun GH Fautsch SK Successful adoptive transfer and in vivo expansion of human haploidentical NK cells in patients with cancer. Blood (2005) 105(8):3051–7. 10.1182/blood-2004-07-2974 15632206

[12] Koehl U Brehm C Huenecke S Zimmermann SY Kloess S Bremm M Clinical grade purification and expansion of NK cell products for an optimized manufacturing protocol. Front Oncol (2013) 3:118. 10.3389/fonc.2013.00118 23730623 PMC3656406

[13] Hayakawa Y Smyth MJ. CD27 dissects mature NK cells into two subsets with distinct responsiveness and migratory capacity. J Immunol (2006) 176(3):1517–24. 10.4049/jimmunol.176.3.1517 16424180

[14] Chiossone L Chaix J Fuseri N Roth C Vivier E Walzer T. Maturation of mouse NK cells is a 4-stage developmental program. Blood (2009) 113(22):5488–96. 10.1182/blood-2008-10-187179 19234143

[15] Podgorny PJ Liu Y Dharmani-Khan P Pratt LM Jamani K Luider J Immune cell subset counts associated with graft-versus-host disease. Biol Blood Marrow Transplant (2014) 20(4):450–62. 10.1016/j.bbmt.2014.01.002 24406506

[16] Chang YJ Zhao XY Huang XJ. Effects of the NK cell recovery on outcomes of unmanipulated haploidentical blood and marrow transplantation for patients with hematologic malignancies. Biol Blood Marrow Transplant (2008) 14(3):323–34. 10.1016/j.bbmt.2007.12.497 18275899

[17] Abrahamsen IW Somme S Heldal D Egeland T Kvale D Tjonnfjord GE. Immune reconstitution after allogeneic stem cell transplantation: the impact of stem cell source and graft-versus-host disease. Haematologica (2005) 90(1):86–93. 15642674

[18] Kollman C Howe CW Anasetti C Antin JH Davies SM Filipovich AH Donor characteristics as risk factors in recipients after transplantation of bone marrow from unrelated donors: the effect of donor age. Blood (2001) 98(7):2043–51. 10.1182/blood.V98.7.2043 11567988

[19] de Koning C Plantinga M Besseling P Boelens JJ Nierkens S. Immune reconstitution after allogeneic hematopoietic cell transplantation in children. Biol Blood Marrow Transplant (2016) 22(2):195–206. 10.1016/j.bbmt.2015.08.028 26341398

[20] Filipovich AH Weisdorf D Pavletic S Socie G Wingard JR Lee SJ National Institutes of Health consensus development project on criteria for clinical trials in chronic graft-versus-host disease: I. Diagnosis and staging working group report. Biol Blood Marrow Transplant (2005) 11(12):945–56. 10.1016/j.bbmt.2005.09.004 16338616

[21] Klyuchnikov E Asenova S Kern W Kilinc G Ayuk F Wiedemann B Post-transplant immune reconstitution after unrelated allogeneic stem cell transplant in patients with acute myeloid leukemia. Leuk Lymphoma (2010) 51(8):1450–63. 10.3109/10428194.2010.496015 20557144

[22] Bae KW Kim BE Koh KN Im HJ Seo JJ. Factors influencing lymphocyte reconstitution after allogeneic hematopoietic stem cell transplantation in children. Korean J Hematol (2012) 47(1):44–52. 10.5045/kjh.2012.47.1.44 22479277 PMC3317470

[23] Charrier E Cordeiro P Brito RM Mezziani S Herblot S Le Deist F Reconstitution of maturating and regulatory lymphocyte subsets after cord blood and BMT in children. Bone Marrow Transplant (2013) 48(3):376–82. 10.1038/bmt.2012.176 23064038

[24] Fedoriw Y Samulski TD Deal AM Dunphy CH Sharf A Shea TC Bone marrow B cell precursor number after allogeneic stem cell transplantation and GVHD development. Biol Blood Marrow Transplant (2012) 18(6):968–73. 10.1016/j.bbmt.2012.03.005 22446015 PMC3693471

[25] Shono Y Ueha S Wang Y Abe J Kurachi M Matsuno Y Bone marrow graft-versus-host disease: early destruction of hematopoietic niche after MHC-mismatched hematopoietic stem cell transplantation. Blood (2010) 115(26):5401–11. 10.1182/blood-2009-11-253559 20354171

[26] Podgorny PJ Pratt LM Liu Y Dharmani-Khan P Luider J Auer-Grzesiak I Low counts of B cells, natural killer cells, monocytes, dendritic cells, basophils, and eosinophils are associated with postengraftment infections after allogeneic hematopoietic cell transplantation. Biol Blood Marrow Transplant (2016) 22(1):37–46. 10.1016/j.bbmt.2015.09.003 26363444

[27] Przepiorka D Weisdorf D Martin P Klingemann HG Beatty P Hows J 1994 consensus conference on acute GVHD grading. Bone Marrow Transplant (1995) 15(6):825–8. 7581076

