# Supplementary material for: Relation between Acute GVHD and NK Cell Subset Reconstitution Following Allogeneic Stem Cell Transplantation
Source: Front Immunol. 2016 Dec 22;7:595. doi: 10.3389/fimmu.2016.00595 (PMC5177660; doi:10.3389/fimmu.2016.00595)
Supplement: Supplementary file 1 [file data_sheet_1.pdf]

Relation between acute GVHD and NK cell subset reconstitution following allogeneic stem cell transplantation

Evelyn Ullrich, Emilia Salzmann-Manrique, Shahrzad Bakhtiar et al. 2016

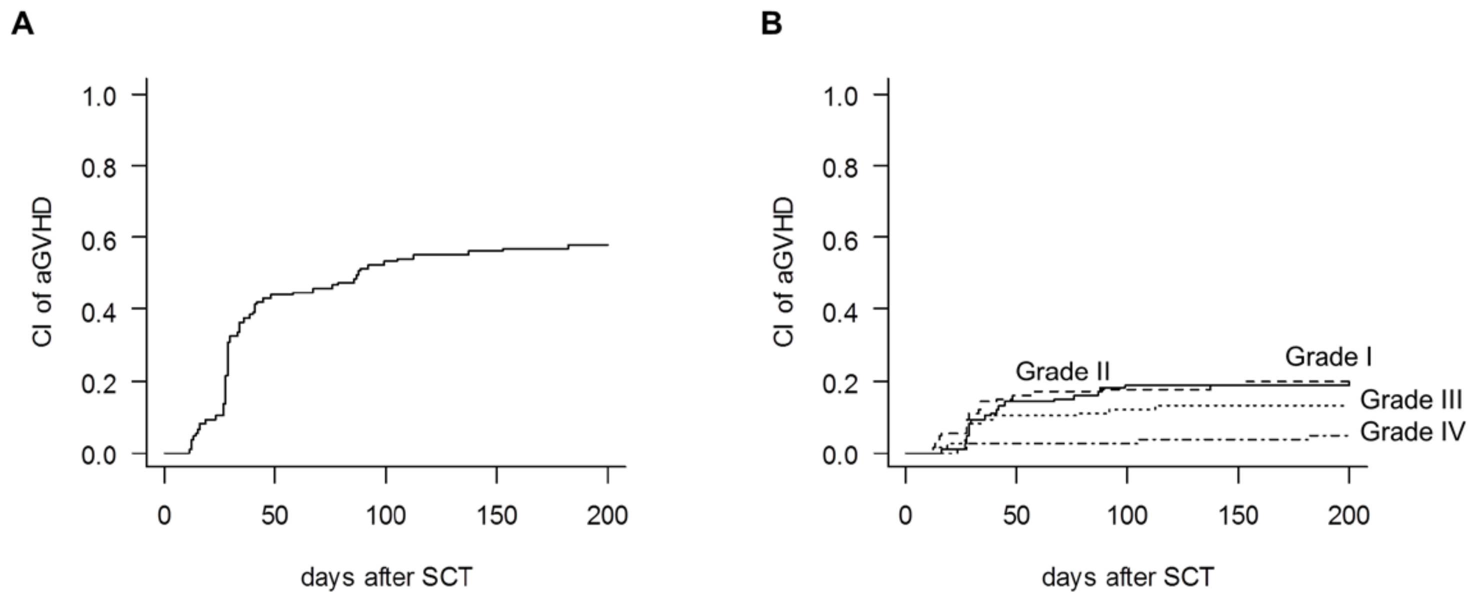

Supplementary Figure 1. Cumulative incidence of aGVHD.

The plot shows the cumulative incidence (CI) of developing any grade of aGVHD during the first 200 days after SCT (**A**) and the CI curve according to the severity of aGVHD (grade I-IV) (**B**).

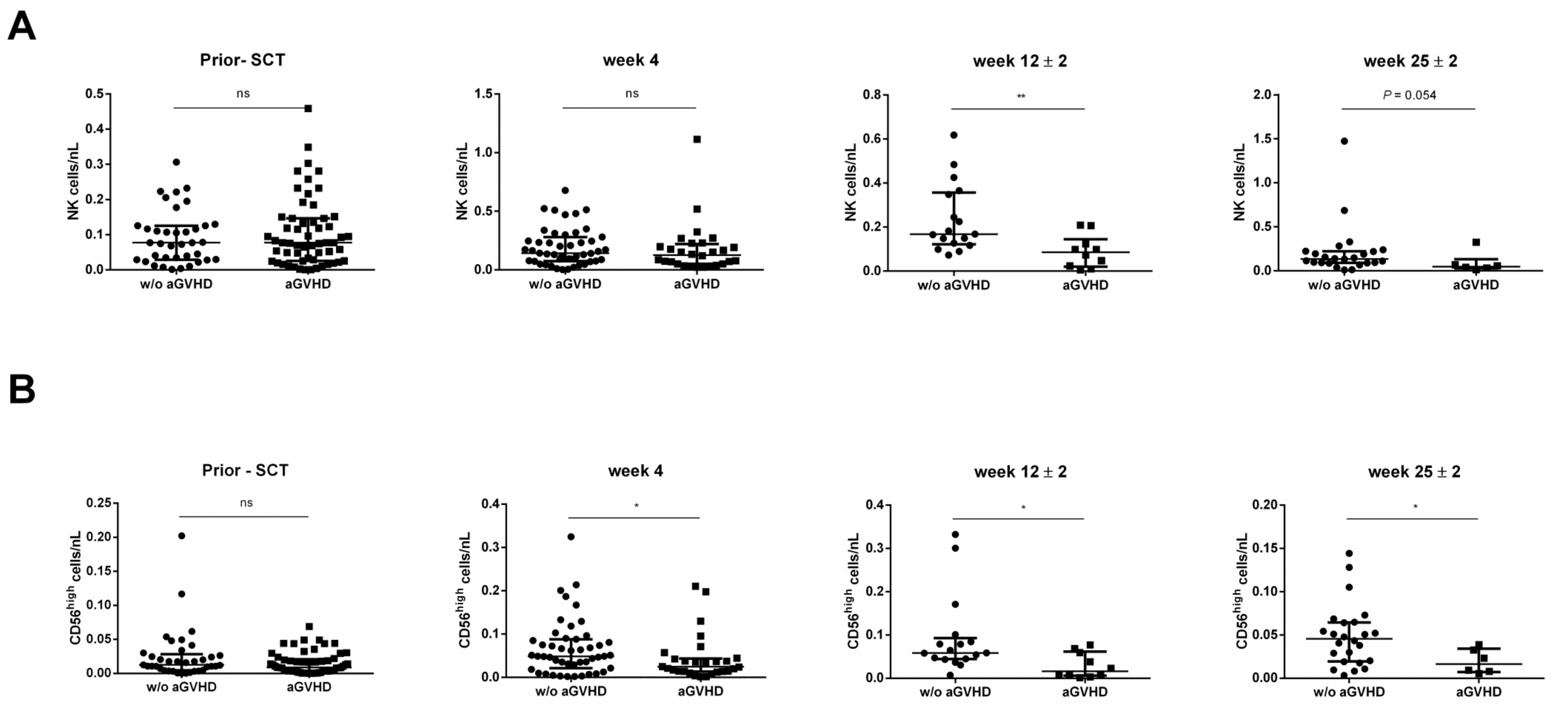

Supplementary Figure 2. NK cell numbers at defined time points in patients after allo-SCT. The number of NK cells **(A)** or specific the $CD56^{high}$ NK cell subset **(B)** in patients without (w/o) compared to patients with aGVHD is analysed by two-sided non-parametric Mann Whitney Wilcoxon Test prior to SCT and at given time points at the routinely performed outpatients visits around week 4, 12 and 25 after allo-SCT. * $p < 0.05$; ** $p < 0.001$; ns=not significant.

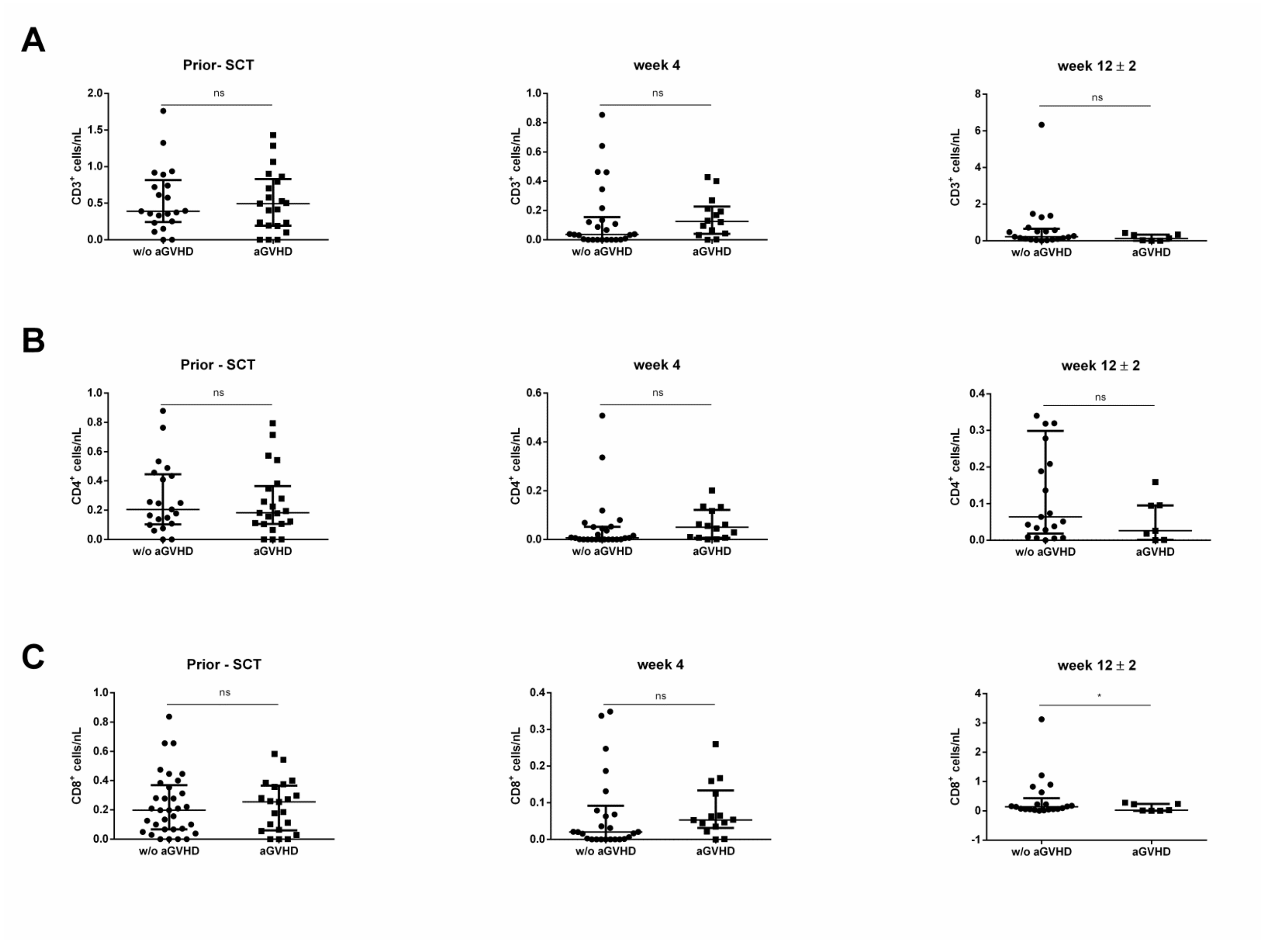

Supplementary Figure 3. T cell numbers at defined time points in patients after allo-SCT. The number of $CD3^+$ T cells **(A),** $CD4^+$ T cells **(B)** or $CD8^+$ T cells **(C)** in patients without (w/o) compared to patients with aGVHD is analysed by two-sided non-parametric Mann Whitney Wilcoxon Test prior to SCT and at given time points at the routinely performed outpatients visits around week 4, and 12 after allo-SCT. * $p < 0.05$; ns=not significant.

Supplementary Table 1.

Impact of steroids on NK cell subset distribution

The table shows the beta coefficients and corresponding P-values for each stage of disease at the given time point for all patients who developed aGVHD later during the observation period of 200 days after SCT. The beta coefficients are given in log10 scale due to previous log10-transformed NK cell subsets.

	Beta (SE)	P-value
**NK cells**		
before aGVHD, ST	-0.366 (0.17)	0.032
during aGVHD, no ST	-0.171 (0.14)	0.220
during aGVHD; ST	-0.436 (0.12)	<0.001
**$CD56^{high}$**		
before aGVHD, ST	-0.412 (0.18)	0.028
during aGVHD, no ST	-0.241 (0.152)	0.114
during aGVHD; ST	-0.548 (0.13)	<0.001
**$CD56^{dim}$**		
before aGVHD, ST	-0.403 (0.18)	0.030
during aGVHD, no ST	-0.189 (0.15)	0.215
during aGVHD; ST	-0.423 (0.13)	0.001

Abbreviations: NK cells, natural killer cells; $CD56^{high}$, immature $CD14^{-}CD3^{-}CD56^{high}CD16^{dim}$ NK cells; $CD56^{dim}$, mature $CD14^{-}CD3^{-}CD56^{dim}CD16^{high}$ NK cells; aGVHD, acute graft-versus-host disease; Beta, beta coefficient; SE, standard error; ST, steroid treatment.
